# Data Consistency and Interpretation of Interleukin-6 and Cardiovascular Outcomes in MESA

**DOI:** 10.1016/j.jacadv.2026.102800

**Published:** 2026-05-14

**Authors:** Kimie Tanaka, Masataka Sata

**Affiliations:** a Juntendo University Graduate School of Medicine, Tokyo, Japan; b Institute of Biomedical Sciences, Tokushima University Graduate School, Tokushima, Japan

We read with great interest the study by Khan et al.^1^ exploring the association between elevated interleukin (IL)-6 levels and cardiovascular disease (CVD) outcomes in the MESA (Multi-Ethnic Study of Atherosclerosis) cohort. Although the study provides valuable insights, we identified several numerical discrepancies and points of clarification that may impact the interpretation of the findings.

First, regarding all-cause mortality for tercile 3, the abstract and the main text report an adjusted HR of 1.98 (95% CI: 1.67-2.36) in model 2, whereas Supplemental Table 1 and Figure 1 list it as 1.86 (95% CI: 1.54-2.25).

Second, the results for incident heart failure (HF) appear inconsistent. The Results section mentions an adjusted HR of 0.80 (95% CI: 0.45-1.45), suggesting the absence of increased risk. In contrast, the abstract, Figure 1, and Supplemental Tables report a contradictory HR of 1.48 (95% CI: 0.99-2.19) for IL-6 tercile 3 in model 2. Furthermore, within Figure 1 and Supplemental Tables, the HR is presented as 1.11 (95% CI: 1.01-1.22) when treated as a continuous variable, shifting the finding to statistical significance. In addition to these discrepancies, the competing risk analysis in Supplemental Table 2 lists an HR of 1.10 (95% CI: 0.90-1.36) for IL-6 tercile 3 in model 2. These divergent values—ranging from no increased risk to a substantial risk increase—make it difficult to ascertain the true association between IL-6 and HF in this study.

Third, there is a potential misalignment between the Results and the Discussion. The Discussion and the Central Illustration state that IL-6 was "consistently associated" with all major CVD outcomes. Conversely, the Results section notes that "the risk of HF with CVD did not increase with increasing IL-6 levels." Clarification on whether HF is considered part of this consistent association would be beneficial.

Fourth, in the subgroup analysis for Hispanic participants, the text cites a *P* value of 0.14 for the model 2 analysis of tercile 1. However, Table 2 lists this interaction *P* value as 0.16, whereas 0.14 appears under tercile 3 (model 1).

Lastly, a minor typographical error was noted in the column header of Supplemental Table 1, which mentions "100,000 person-years." The table title, the Methods section, and the raw data indicate that "1,000 person-years" is the correct unit.

We appreciate the authors’ contribution to understanding the role of chronic inflammation in cardiovascular risk prediction across all racial and ethnic groups, and we look forward to their clarification to ensure a deeper understanding of these significant findings.

## References

[1] Khan M.S., Talha K.M., Maqsood M.H. (2024). Interleukin-6 and cardiovascular events in healthy adults: MESA. JACC Adv.

